# Impact of Point-of-Care Testing on Diagnosis, Treatment, and Surveillance of Vaccine-Preventable Viral Infections

**DOI:** 10.3390/diagnostics15020123

**Published:** 2025-01-07

**Authors:** Kirthika Lakshmanan, Benjamin M. Liu

**Affiliations:** 1 College of Osteopathic Medicine, Kansas City University, Manhattan, KS 66506, USA; kirthika.lakshmanan@kansascity.edu; 2 Division of Pathology and Laboratory Medicine, Children’s National Hospital, Washington, DC 20010, USA; 3 Department of Pediatrics, George Washington University School of Medicine and Health Sciences, Washington, DC 20010, USA; 4 Department of Pathology, George Washington University School of Medicine and Health Sciences, Washington, DC 20037, USA; 5 Department of Microbiology, Immunology & Tropical Medicine, George Washington University School of Medicine and Health Sciences, Washington, DC 20037, USA; 6 Children’s National Research Institute, Washington, DC 20012, USA; 7 The District of Columbia Center for AIDS Research, Washington, DC 20052, USA

**Keywords:** point-of-care testing, vaccine-preventable infections, viral diseases, CRISPR-Cas, viral load, HBV, mpox, dengue, influenza, respiratory viruses, mutations, resistance

## Abstract

With the advent of a variety of vaccines against viral infections, there are multiple viruses that can be prevented via vaccination. However, breakthrough infections or uncovered strains can still cause vaccine-preventable viral infections (VPVIs). Therefore, timely diagnosis, treatment, and surveillance of these viruses is critical to patient care and public health. Point-of-care (POC) viral diagnostics tools have brought significant improvements in the detection and management of VPVIs. These cutting-edge technologies enable prompt and accurate results, enhancing patient care by facilitating timely treatment decisions. This review delves into the advancements in POC testing, including antigen/antibody detection and molecular assays, while focusing on their impact on the diagnosis, treatment, and surveillance of VPVIs such as mpox, viral hepatitis, influenza, flaviviruses (dengue, Zika, and yellow fever virus), and COVID-19. The role of POC tests in monitoring viral infection is crucial for tracking disease progression and managing outbreaks. Furthermore, the application of POC diagnostics has shown to be vital for public health strategies. In this review, we also highlight emerging POC technologies such as CRISPR-based diagnostics and smartphone-integrated POC devices, which have proven particularly beneficial in resource-limited settings. We underscore the importance of continued research to optimize these diagnostic tools for wider global use for mpox, viral hepatitis, influenza, dengue, and COVID-19, while also addressing current challenges related to their sensitivity, specificity, availability, efficiency, and more.

## 1. Introduction

Vaccine-preventable viral infections (VPVIs) such as hepatitis B, mpox, dengue, and various respiratory viruses such as influenza viruses pose serious risks to global health due to their potential for severe outcomes and wide transmission [1]. For example, hepatitis B virus (HBV) can lead to chronic liver conditions such as cirrhosis and primary hepatocyte cancer [1]. Respiratory viruses, notably influenza, can cause significant respiratory distress and are capable of precipitating widespread epidemics. Flaviviruses, e.g., dengue fever, can progress from mild symptoms to fatal hemorrhagic fever. The ability of these viruses to mutate contributes to their threat, as new variants may resist current treatments and evade vaccine-induced immunity or antiviral therapy [2,3,4,5,6,7,8]. Thanks to vaccinology, numerous effective vaccines have been developed [9]. HBV vaccines are widely administered worldwide, providing up to 95% protection and reducing the incidence of severe liver disease. The JYNNEOS vaccine, effective against both mpox and smallpox, is crucial for controlling outbreaks. For dengue, Dengvaxia is used in endemic regions to prevent severe forms of the disease in those previously infected. Seasonal influenza vaccines are updated annually to match the most common circulating strains, crucial for protecting populations, especially the vulnerable [9]. Despite these advancements, breakthrough infections illustrate that no vaccine is foolproof. Factors such as antigenic drift, waning immunity, and incomplete vaccination coverage necessitate continuous monitoring and updating of vaccines to maintain their efficacy against emerging strains and ensure public health safety [10].

The detection of VPVIs is crucial not only for providing diagnosis, initiating treatment, and preventing transmission but also for monitoring the effectiveness of vaccines and the emergence of drug-resistant strains. Effective surveillance systems can provide early warnings of outbreaks, facilitate rapid public health responses, and inform necessary updates to vaccine formulations to combat newly emerging strains [2]. Moreover, detecting these infections helps in assessing the real-world efficacy of vaccines post-deployment, guiding future vaccination strategies and public health policies. The ongoing battle against VPVIs underscores the importance of continual advancements in vaccine technology, robust detection methods, and comprehensive healthcare strategies to mitigate the impacts of these significant viral threats [1,2]. The effectiveness of detecting VPVIs can be significantly influenced by the methodologies employed, primarily centralized versus point-of-care (POC) testing. On the one hand, centralized testing, typically conducted in well-equipped laboratories, offers comprehensive diagnostic capabilities with high throughput, which is essential for large-scale surveillance and detailed virological studies. However, the logistics of sample transportation and the time required for processing and analysis can delay the response to outbreaks [11]. On the other hand, POC testing, which involves conducting tests at or near the site of patient care, offers rapid results that are crucial for immediate clinical decision-making and public health interventions. POC testing simplifies the diagnostic process, reduces turnaround times, and is increasingly accessible in diverse settings, from urban hospitals to remote areas. This shift towards POC testing is transforming outbreak management by enabling quicker containment and more adaptive responses to the dynamic nature of VPVI threats [11,12].

POC testing plays a pivotal role in modern healthcare by enabling rapid, on-site clinical laboratory tests. These tests are conducted close to the patient, providing timely diagnosis and facilitating prompt treatment [12]. POC testing has been particularly vital in managing infectious diseases, where the rapid identification and differentiation of viral agents have significantly improved patient outcomes, case isolation, and disease prevention. The technologies developed for POC testing have extended their applications to manage a wide range of VPVIs, including influenza, hepatitis, and mpox. These tests have proven instrumental in enabling early and accurate diagnoses, allowing healthcare providers to prescribe appropriate treatments more efficiently. Advances in technology, such as miniaturizing electronic components and improving diagnostic instruments, have further enhanced the portability and precision of POC devices [12,13]. Additionally, POC testing empowers healthcare professionals to monitor chronic patients closely and adjust clinical management rapidly when necessary, ensuring optimal care [14].

Despite the advancements in POC testing for VPVIs, there remain several challenges that limit its broader application. Knowledge gaps in standardized protocols and various types of POC technologies across diverse populations and healthcare settings present significant barriers [1]. Technical issues, such as the sensitivity and specificity of POC assays, often vary based on the viral agent and patient demographics, leading to potential diagnostic inaccuracies in certain populations [15]. Regulatory challenges also arise, as POC tests must meet stringent quality standards and gain approval across different countries, which can delay their availability in emerging markets [15]. This review article sheds light on the current innovations, evaluates ongoing challenges, and explores potential future directions for enhancing the reach and efficacy of POC technologies for VPVIs. By addressing these barriers, we provide a perspective on employing POC testing for more equitable and efficient diagnostic solutions that strengthen global health resilience.

## 2. Progress in POC Technologies

**Figure 1:**
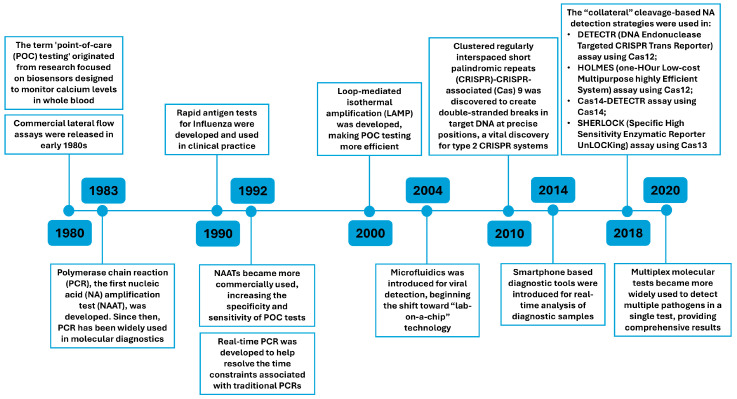
Timeline of various point-of-care (POC) technologies. This timeline illustrates key advancements in POC testing technology in the past four decades. Each milestone represents a significant development in diagnostic tools and methods that has contributed to the increasing efficiency, accuracy, and accessibility of POC testing in various healthcare settings [11,12,13,17,18,19,20].

Recent advancements in POC technology have revolutionized the detection of viral infectious diseases, particularly viruses that are vaccine preventable. These innovations in diagnostic tools are expected to overcome the limitations of conventional laboratory procedures, such as slow turnaround times and inadequate sensitivity and specificity in responding to emerging viral outbreaks [11,12,16]. Technological progress has introduced innovative approaches such as biosensors, clustered regularly interspaced short palindromic repeats (CRISPR), CRISPR-associated proteins (Cas), and smartphone-enabled diagnostics [17]. As shown in Figure 1, these technologies have evolved significantly over time. New technologies have enabled healthcare practitioners to deliver precise and rapid results at the point of care, greatly improving patient outcomes. Currently, molecular POC testing for viruses is widely used for various viral illnesses, including COVID-19, HIV, and hepatitis. With future advancements, such as CRISPR-based systems and the integration of smartphones with molecular POC devices, the future of POC testing is promising [13,17].

**Figure 2:**
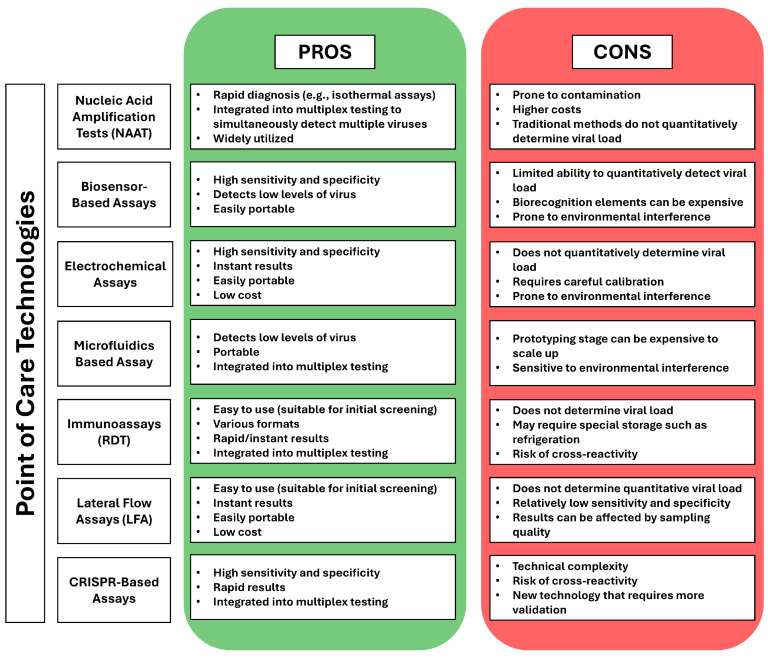
This chart compares the advantages and disadvantages of different point-of-care (POC) technologies used to detect VPVIs. Each technology has unique characteristics that make it suitable for specific applications in clinical and field settings [11,12,13,20,21,23].

Advancements in POC technologies have significantly expanded the market with a variety of diagnostic kits designed for detecting respiratory viruses. These kits increasingly incorporate molecular biology techniques alongside lateral flow assays (LFA), microfluidics, and enzyme-linked immunosorbent assay (ELISA) formats, all aimed at improving the sensitivity and efficiency of viral detection [16,17]. By enabling highly sensitive tests to be conducted directly at the point of care, these technologies ensure that patients in remote or underserved areas can receive timely diagnosis and treatment, reducing delays in care [12,13,16]. Multiple different types of POC assays and their advantages and disadvantages are highlighted in Figure 2. The chart compares the strengths and limitations of various POC technologies used for VPVI diagnostics. Technologies such as NAATs, biosensor-based assays, electrochemical assays, microfluidics, immunoassays, LFAs, and CRISPR-based assays each offer advantages, including high sensitivity, rapid results, and portability, making them suitable for quick, decentralized testing [11,12,13,20,21]. However, they also present drawbacks, such as susceptibility to contamination, environmental interference, limited quantitative capabilities, and, in some cases, high costs [11,12,13,20,21,22]. While each method has unique benefits, its limitations must be managed to ensure effective and reliable use in various healthcare settings.

### 2.1. Advancements in Nucleic Acid Amplification Tests (NAATs)

In contrast to traditional culture methods, NAAT techniques have multiple advantages that make them more suitable for the rapid and sensitive identification of pathogens, which are widely used in research and clinical settings [24,25,26,27,28,29,30]. PCR and isothermal amplification are essential for virus detection in POC settings [11,12,13,17,18,19,20]. NAATs, such as loop-mediated isothermal amplification (LAMP) and recombinase polymerase amplification (RPA), offer significant benefits in POC settings [17]. These technologies eliminate the need for complex thermal cycling, as required by traditional PCR, allowing for faster and more portable diagnostics. LAMP, for instance, amplifies nucleic acids at a constant temperature [17]. However, NAATs can be susceptible to contamination, which may lead to false positives [11,12]. In addition, there is a need for specialized reagents that can increase costs and limit accessibility. Despite these challenges, innovations such as CRISPR-based NAATs aim to improve specificity and reduce error rates, making these technologies even more suitable for POC settings [12,13,17].

### 2.2. Biosensors

Biosensors represent another significant advancement in POC technology, enabling real-time monitoring of viral particles. Biosensors are sophisticated diagnostic devices composed of tightly integrated biological and physicochemical layers that work together to detect specific viral biomarkers, such as proteins, antigens, or nucleic acids [31,32]. These layers include a biorecognition element, which binds to the target biomarker, and a transducer that converts the biological interaction into an electrical, optical, or electrochemical signal [32]. The innovative design of these biosensors allows for high sensitivity and specificity in identifying pathogens. By incorporating nanomaterials and enabling early advanced signal amplification techniques, modern biosensors can detect minute quantities of viral particles and provide accurate diagnoses even where there is a low viral quantity present in a sample [33]. In the context of POC diagnostics, biosensors excel at providing rapid results in a wide range of settings. Unlike conventional laboratory testing, which requires extensive equipment and specialized personnel, biosensor-based POC devices are compact, portable, and user-friendly, making them accessible for use in non-clinical environments [32,33]. This technology is particularly valuable in high-risk areas, such as airports, schools, and public transportation, where quick detection of viral infections is critical for minimizing transmission [17,31]. By identifying infected individuals early, biosensors not only support timely treatment but also help contain outbreaks by reducing the spread of infectious diseases.

There are numerous bioanalytical approaches that biosensors employ for analyte detection. Each bioanalytical approach utilizes specific bioreceptors, for example, enzymes, antibodies, DNA probes, or whole cells, to enable the conversion of the biomolecular interaction into a measurable signal and selective detection of target analytes in a biological sample [33,34]. First, the electrochemical (e.g., amperometric, potentiometric, or impedimetric) approach has multiple advantages over nonelectrochemical approaches, e.g., rapidity, low cost, high sensitivity, and broad application for a long list of viruses [33]. For example, Devarakonda et al. [35] developed a handmade, disposable electrochemical immunosensor enabling rapid detection of influenza virus H1N1 with excellent selectivity. Second, optical methods (e.g., fluorescence and surface plasmon resonance) have been used in biosensors for viral detection. For example, three categories of potential optical biosensors including spectroscopic-, nanomaterial-, and interferometry-based approaches have been developed for detecting various types of viruses, including SARS-CoV-2 [34]. Third, there are also mass-based approaches (piezoelectric). For example, Guliy et al. developed a piezoelectric resonator-based sensor system coupled with a lateral electric field in the frequency range of 6–7 MHz of the electric field that enables virus detection [36].

While biosensors offer high sensitivity and rapid detection for POC viral diagnostics, they also carry several limitations. One major drawback is their requirement of highly specific biorecognition elements, which are expensive and challenging to produce. Furthermore, biosensors can be prone to environmental interference, such as temperature fluctuations or sample impurities, which can also affect their accuracy [37]. Additionally, the miniaturization and integration of biosensors into user-friendly devices still require technical improvement, particularly for use in resource-limited settings [17,31,32].

The other major advancement in POC technologies is the incorporation of such technology into smart devices. These systems use optical and electrochemical biosensing approaches to detect biomolecules, with smartphones interpreting test results in real time. For instance, optical biosensing employs a smartphone’s camera to analyze color changes in tests, while electrochemical biosensing relies on the smartphone to measure electrical signals from biochemical reactions [38]. One novel example of such a technology is the first successful detection of dengue virus using a smartphone-based biosensor called Portronicx. This technology combines a wireless Potentiostat with a smartphone interface [39]. A Potentiostat is an electronic device that controls the voltage between electrodes in an electrochemical cell while measuring the resulting current, often used to study chemical reactions or detect specific substances in sensors [39]. This portable platform uses a three-electrode setup and an Android app to dengue antigen within 20 s with high sensitivity. The system’s miniaturization offers affordability, portability, and design flexibility, showcasing strong potential for commercialization in mobile healthcare diagnostics [13,17,39].

One of the key advantages of these smartphone-based systems is their ability to connect to cloud platforms, facilitating real-time data sharing and monitoring, which is particularly valuable during public health emergencies [17,21]. This integration proved highly effective during the COVID-19 pandemic, allowing widespread testing outside of clinical settings and easing the burden on centralized laboratories [21]. The scalability and accessibility of smartphone-integrated POC testing also make it a powerful tool for global health and epidemiological monitoring. By enabling remote, real-time data collection and analysis, these systems can track disease spread, monitor chronic conditions, and assess treatment outcomes across different regions, including resource-limited areas [21,39]. This technology offers significant benefits for public health, particularly in underserved populations, where access to traditional healthcare infrastructure may be limited [17,21,39]. The portability, affordability, and ease of use of smartphone-based diagnostics make them well-suited for large-scale usage.

### 2.3. CRISPR-Cas

One of the most groundbreaking technologies applied to POC diagnostics is the CRISPR-Cas technique for virus detection. Initially developed for gene editing, CRISPR-Cas has been repurposed for diagnostics due to its ability to provide highly sensitive and specific detection of viral nucleic acid sequences [40]. CRISPR-Cas systems function by utilizing guide RNA sequences that are designed to bind specifically to complementary viral nucleic acid sequences [41]. When the guide RNA binds to its target, the various Cas enzymes create a break in the nucleic acid [40,41]. This cleavage event can trigger a detectable signal that is picked up by diagnostic instruments. Optical analytical signals, e.g., fluorescence, are typically employed in detection systems based on CRISPR-Cas. The mechanism of the CRISPR-Cas system allows for a highly sensitive and specific detection of viral pathogens. CRISPR-based diagnostic tools, such as CRISPR-Cas12 and CRISPR-Cas13, have already been successfully employed for the detection of viruses including SARS-CoV-2, with promising potential for identifying emerging viruses in the future [12,40,41]. In addition to being sensitive and specific, these systems are fast, cost-effective, and can be adapted to detect a wide range of pathogens [41]. Incorporating CRISPR technology into POC testing allows for instant results, even in settings with limited facilities and resources. Moreover, the capability to target a wide range of viral sequences makes CRISPR diagnostics valuable in the massive scope that is the fight against viral threats [40,41].

While CRISPR-Cas technology holds great promise for POC viral diagnostics due to its high specificity and sensitivity, there are several limitations that may affect its practicality. One key challenge is the need for precise conditions, including temperature control, to ensure optimal function of the Cas enzymes and guide RNAs [42]. Additionally, CRISPR diagnostics often require pre-amplification of target nucleic acids, which increases the complexity and time required for testing, requiring further development to make it more suitable for rapid, on-site diagnostics [22]. Furthermore, the cost of reagents and the technical expertise needed for sample preparation and reaction control pose barriers to widespread use in low-resource areas [22,41]. There are also noteworthy issues in the accuracy of the assay. There are reported causes of false positives due to off-target effects, where the guide RNA binds to non-target sequences [22,41,42]. This limits their reliability in critical diagnostics, especially when high specificity is required, such as in distinguishing between closely related viral strains [42]. While innovations are underway to simplify these systems and improve their usability in field settings, these challenges must be addressed to realize the full potential of CRISPR-based POC diagnostics [40,41,42].

## 3. Impact of Viral POC Testing on Diagnosis and Treatment of VPVIs

Generally, molecular testing can be categorized into qualitative and quantitative tests, each serving different purposes in diagnostics. Qualitative molecular tests are designed to detect the presence or absence of a specific pathogen’s genetic material, providing a simple “yes or no” result [43]. These tests are particularly useful for confirming the diagnosis of an infection. Quantitative molecular tests measure the specific amount of viral genetic material present in a sample, providing insight into viral load (VL). Quantitative assays are essential for monitoring the severity of an infection, tracking disease progression, and assessing the effectiveness of treatment, especially in chronic infections such as HBV or HIV [43,44]. POC has applications in both types of assays. POC viral tests and VL assays have revolutionized healthcare, particularly in the management of VPVIs [12]. Including VL testing in point-of-care settings for VPVIs is particularly important as it helps assess the infection’s severity, monitor treatment effectiveness, and guide clinical decisions [12,13,17]. By measuring the amount of virus present in a patient’s system, healthcare providers can quickly determine whether a vaccine or treatment is working effectively or if additional interventions are needed. It also aids in identifying patients at higher risk of transmitting the virus, allowing for timely containment and prevention measures. VL testing plays a key role in personalizing patient care, ensuring that decisions are based on precise, real-time information about the infection’s status [12,13].

These tools have significantly improved diagnostic accuracy, which in turn has positively impacted treatment decisions and patient outcomes for various viral infections, including mpox, HBV, influenza, dengue, COVID-19, and enterovirus. A study by Mattila et al. involving a randomized clinical trial on POC testing for respiratory pathogens demonstrated a notable reduction in antibiotic prescriptions for children when rapid diagnostic tests were employed [45]. This is especially relevant in the management of VPVIs such as influenza, where unnecessary antibiotics are often used symptomatically when a definitive diagnosis is unavailable. Accurate identification of viral pathogens prevents the misuse of antibiotics, which are ineffective against viruses and can contribute to antibiotic resistance [12,13,45].

Similarly, Brendish et al. conducted a study among adults with exacerbations of airway diseases and found that POC testing for respiratory viruses not only reduced unnecessary antibiotic prescriptions but also shortened hospital stays and improved patient outcomes [46]. Thus, POC testing, with its rapid turnaround time and accessibility, ensures timely intervention, reduces antibiotic overuse and promotes better resource utilization. This section explores how POC testing and VL assays have contributed to managing viral infections and improving patient care [46].

### 3.1. Mpox

Mpox is caused by an Orthopoxvirus, MPXV, which shares similarities in life cycles and pathogenesis with other Orthopoxviruses [47,48]. However, the recent surge in mpox cases highlights the critical role of POC testing in the diagnosis and management of the disease [49]. Although mpox is a VPVI, it remains highly contagious once contracted [47]. In such cases, rapid diagnosis becomes essential, particularly for immunocompromised patients [49,50]. Skin lesion swabs have emerged as a more sensitive and less invasive diagnostic method compared to blood or cerebrospinal fluid sampling, offering a faster and more accessible means of detecting the virus [49]. Additionally, the detection of the mpox-causing virus, MPXV, from these skin samples provides valuable insights into the severity of the infection, aiding in tailoring treatment approaches [49]. POC mpox testing also allows for the early identification of severe cases, enabling timely interventions and supporting vaccination campaigns for at-risk populations [12,49,50].

POC testing becomes particularly important in light of the development of the new mpox vaccine. The mpox vaccine, JYNNEOS (also known as Imvamune or Imvanex), is a third-generation, non-replicating smallpox vaccine approved for the prevention of both smallpox and mpox [50]. Unlike earlier smallpox vaccines, JYNNEOS is considered safer, especially for immunocompromised individuals, as it does not contain a live virus capable of replication [49,50]. This vaccine has become a critical tool in controlling mpox outbreaks and offers protection for high-risk populations.

One example of mpox detection technology is the Cepheid Xpert mpox POC test (Cepheid, Sunnyvale, CA, USA), which has proven effective in delivering rapid results [51]. It was only one of two POC tests that were approved for emergency use by the U.S. Food and Drug Administration (FDA) in 2023 [50,52]. This test is intended for the qualitative detection of mpox in human lesion swab specimens from individuals suspected of mpox by their healthcare provider in the POC setting. The test takes 36 min to complete and delivers results for two targets: clade IIb-specific mpox and non-variola Orthopoxviruses [51,52]. The test is a real-time PCR test that results in the qualitative detection of DNA in specimens. Xpert has been tested against traditional mpox by the Institut National de Recherche Biomedicale in Kinshasa and was found to have a specificity of 98.8% and sensitivity of 100%, making the test highly reliable in mpox diagnosis [53]. However, Xpert has been found to have limitations, such as the ability to screen only for a single clade (II) of the virus and limited availability in certain regions due to pending FDA clearances [50,53,54].

In addition to the Cepheid technology, the CDC has incorporated its testing from mucosal tissues into its management guidelines, underscoring the importance of POC testing in diagnosing and managing mpox [52,54]. As Suñer et al. emphasized, understanding viral clearance times is crucial for developing effective isolation protocols and ensuring appropriate recovery periods, which protect both patients and the wider community [55]. Despite certain constraints, POC testing remains a vital tool in controlling the spread of mpox, especially in high-risk groups and resource-limited settings.

### 3.2. HBV

HBV remains a major global health challenge, and POC testing plays a pivotal role in early detection, disease monitoring, and improving access to healthcare in underserved areas [14]. Viral assays have been particularly valuable in identifying infection sources and enabling timely interventions, helping to prevent chronic liver conditions such as cirrhosis and liver cancer [1,56,57]. POC testing enhances diagnosis and clinical management, significantly reducing morbidity and mortality among HBV-infected individuals. For HBV, POC testing can also enhance vaccine allocation by identifying unvaccinated individuals or those at a high risk of infection [14,56].

Despite its advantages, a significant limitation of POC testing in developing countries is the geographical disconnect between testing sites and treatment centers [56]. In many regions, patients may receive rapid diagnostic results through POC tests, but the distance to treatment facilities delays access to necessary interventions, such as antiviral medications or further diagnostic follow-ups [14,56]. This separation can be exacerbated by limited infrastructure, leading to delayed treatment, increased disease transmission, and worse health outcomes. Addressing this gap requires improved healthcare integration and resource allocation to bridge diagnostic and treatment services effectively [56,57].

In the United States, HBV diagnosis typically involves laboratory-based tests rather than POC options. The standard diagnostic process includes a blood test to detect hepatitis B surface antigen (HBsAg), which indicates an active HBV infection. Additionally, tests for hepatitis B surface antibody (anti-HBs) and hepatitis B core antibody (anti-HBc) help assess past exposure or immunity [14,58]. If an infection is confirmed, more advanced laboratory tests, such as HBV DNA quantification are used to evaluate the severity of the infection and guide treatment decisions [58]. These tests are usually conducted in clinical laboratories or hospitals rather than at the point of care. Rapid or POC tests are less commonly used for HBV diagnosis in the U.S., which may result in delays in patient care. While Cepheid’s POC testing for HBV VL offers a cost-effective and efficient solution for managing the infection, it has not yet received market approval in the United States [14,59,60].

In addition, blood transfusions are a potential route for transmitting HBV. Therefore, it is vital to screen donated blood for HBV to prevent the spread of the virus to recipients [61]. Even though advanced screening methods are used in most developed countries to ensure the safety of blood supplies, there is still a risk, particularly in regions with limited resources. Transfusion-related transmission of HBV can occur if screening methods fail to detect infections during the “window period” when the virus is present but not yet detectable by certain tests [11,14,58,62]. Therefore, ensuring highly sensitive testing, including consideration of the limitations of POC tests, is crucial to maintaining blood safety and protecting public health [14]. Therefore, though POC testing for HBV may not be currently suitable for transfusion screening due to the need for more comprehensive and sensitive assays, its utility shines in settings where advanced diagnostic infrastructure is lacking [58,62]. In rural or resource-limited areas, where access to centralized laboratories is minimal, POC tests provide a critical diagnostic tool [12,58,60].

Finally, the idea of POC testing aligns with global efforts, such as the United Nations’ goal to eliminate viral hepatitis by 2030. The World Health Organization (WHO) and other global health bodies have set a target to eliminate viral hepatitis as a public health threat by 2030, with the aim of reducing new infections by 90% and hepatitis-related deaths by 65% [53,62]. This global initiative focuses on expanding access to vaccines, scaling up testing efforts, and ensuring that effective treatments, such as antiviral therapies for hepatitis B and curative treatments for hepatitis C, are accessible to those in need [62]. Key strategies include integrating viral hepatitis services into primary healthcare, increasing the availability of POC testing, and enhancing public awareness about prevention and treatment options [53,56,62].

### 3.3. Respiratory Viruses

Respiratory viruses such as influenza, COVID-19, and enteroviruses continue to pose significant global health challenges, especially for high-risk populations such as the elderly, immunocompromised individuals, and children [2,63,64,65,66,67,68]. These viruses are highly contagious and can lead to severe health outcomes [16]. Influenza is a well-known cause of widespread morbidity and mortality, particularly during seasonal outbreaks [2]. Similarly, the COVID-19 pandemic has underscored the critical need for rapid and widely accessible diagnostic tools to effectively manage and limit viral transmission [16]. Enteroviruses can lead to serious illnesses such as viral meningitis and myocarditis, further emphasizing the importance of timely and accurate diagnostics to prevent complications [69]. In all cases, POC testing has become an essential tool in early detection, treatment decision-making, and the reduction of transmission rates [12,13,16].

Several POC technologies are available for the rapid detection of influenza, playing a crucial role in managing seasonal outbreaks. One of the most commonly used methods is the Rapid Influenza Diagnostic Test (RIDT), which detects influenza A and B antigens in respiratory specimens within 10 to 15 min [70]. Although convenient and fast, RIDTs have lower sensitivity compared to molecular methods, which may lead to false negatives [70,71]. Rapid molecular tests, such as the Cepheid Xpert Xpress Flu/RSV and Roche’s Cobas Liat system, utilize nucleic acid amplification techniques like PCR to detect viral RNA with greater accuracy and sensitivity, delivering results in 30 min [71]. Additionally, isothermal amplification tests, such as Abbott’s ID NOW Influenza A & B, use a constant temperature to amplify viral RNA, offering results within 13 min and providing a more portable alternative to traditional PCR methods [72]. Emerging biosensor-based POC devices are also being developed to detect influenza antigens or RNA in real time, potentially making them useful in high-traffic areas such as airports or public spaces [32]. These technologies significantly improve the ability to diagnose and manage influenza quickly and effectively, particularly in settings with limited laboratory resources [12]. POC tests provide quick results, enabling clinicians to make timely decisions about antiviral treatments, thus reducing the unnecessary use of antibiotics and improving patient outcomes [16,70,71,72].

To address these drawbacks of POC testing for influenza, newer technologies such as multiplex NAAT testing have been developed. Platforms such as BioFire FilmArray, and QIAstat respiratory multiplex testing can identify multiple respiratory viruses, including influenza, RSV, and COVID-19, in a single test [71]. While these tests enhance diagnostic accuracy, they provide qualitative rather than quantitative results, limiting their use in more vulnerable populations such as transplant recipients [71,72]. For these individuals, more detailed tests, such as RT-PCR or viral culture, may still be necessary to guide treatment decisions [70,73]. Performance characteristics of commonly used multiplex respiratory molecular panels and their clinical applications were recently reviewed in a review article by Contes and Liu [74], including BioFire Respiratory Panel 2.1, BioFire Spotfire, Roche Cobas (GenMark) ePlex respiratory pathogen panel 2 (RP2) panel, QIAstat-Dx Respiratory Panel Plus, and Luminex NxTAG Respiratory Pathogen Panel.

The COVID-19 pandemic underscored the urgent need for widespread and rapid POC testing, especially in emergency and outpatient settings [16]. The accessibility, ease of use, and rapid results provided by POC PCR tests have been essential in reducing the time needed for isolation and treatment, thereby enhancing patient management during critical periods of infection [12,16]. For example, Fistera et al. demonstrated that performing POC PCR tests in emergency departments significantly shortened the time to diagnosis and isolation, which improved overall patient outcomes [75]. The NIH’s Home Test to Treat pilot program further expanded the role of POC testing by allowing individuals to self-diagnose and access treatments for COVID-19 without visiting a healthcare facility, particularly in rural or underserved areas [76].

During the COVID-19 pandemic, POC testing played a crucial role in identifying “super-spreaders”; individuals with extremely high viral quantities who were responsible for transmitting the virus to a disproportionately large number of people [77]. Super-spreaders posed a significant risk in environments such as healthcare facilities, workplaces, and public transportation, where the virus could spread rapidly [77]. POC testing aided in identifying these individuals and enabled immediate isolation and targeted public health interventions. Additionally, POC testing helped in tracking how infectious individuals remained over time, aiding in decisions about the appropriate length of quarantine and the effectiveness of treatment measures [75,77]. For example, Cepheid’s Xpert Xpress SARS-CoV-2 test is a rapid molecular test using PCR technology that can detect and quantify the virus within 30 to 45 min [78]. Similarly, Abbott’s ID NOW system uses isothermal amplification to rapidly detect COVID-19 in as little as 13 min, enabling healthcare professionals to quickly identify super-spreaders [79]. Identifying COVID-19 super-spreaders quickly through POC tests was critical in mitigating transmission, particularly in high-density settings, ultimately helping to control the spread of the virus more effectively [16,77,79].

Despite these advances, POC testing for COVID-19 has limitations. While they offer faster results, some rapid antigen tests have low sensitivity and may fail to detect all positive cases, particularly in individuals with lower VL or early in the infection course [80]. False negatives have been documented, which could lead to missed diagnoses and delayed isolation of infected individuals [11,80]. In addition, home testing often leads to sampling errors or human mistakes, which can contribute to false negatives and reduce the accuracy of the test results [79,80]. Due to these limitations, POC antigen tests should be used in conjunction with more sensitive molecular tests, like PCR, especially in high-risk settings or for confirming negative results in symptomatic patients [79]. This combined approach ensures more accurate diagnosis and supports more informed treatment decisions, ultimately improving patient outcomes and public health strategies [79,80].

Advancements in POC testing, particularly through CRISPR technology, have significantly improved the management of severe diseases and complications caused by enteroviruses, such as viral meningitis and myocarditis [81]. Meningitis and encephalitis caused by enteroviruses can develop rapidly, often within a few days of exposure [82]. Therefore, timely diagnosis can help in preventing these fatal complications. This rapid and highly specific mechanism delivers results in minutes and thus allows healthcare providers to quickly identify various enterovirus strains on-site, without the need for complex lab-based equipment. As a result, tailored treatment approaches can be initiated more quickly, such as administering antiviral medications or specific supportive care measures [35,81,82,83].

Additionally, POC tests equipped with CRISPR technology can detect viruses, giving healthcare providers crucial information about the severity of the infection [40,41]. By measuring the amount of viral RNA present in a patient’s sample, these tests enable clinicians to assess how aggressively the virus is replicating in the body [12]. This information becomes invaluable for triaging patients, allowing for more precise management based on the intensity of the infection. For instance, a patient with a lower VL may be treated with supportive care on an outpatient basis, whereas those with higher VLs might require closer monitoring, antiviral therapy, or intensive neurological care in the case of encephalitis [84]. Furthermore, tracking VL over time through serial POC testing allows healthcare providers to monitor how well the patient is responding to treatment, adjusting care strategies if necessary [82]. This real-time data can also aid in determining when it is safe to discharge a patient or end isolation, reducing unnecessary hospital stays and improving resource allocation during outbreaks [85]. In outbreak scenarios, rapid POC testing helps curb transmission by quickly identifying infected individuals and enabling timely isolation measures [82,85]. This is particularly critical in preventing widespread transmission in settings such as schools or pediatric hospitals, where enteroviruses can spread rapidly [82]. POC testing can also guide allocation of vaccines. Although there is no universal vaccine for all enteroviruses, vaccines for specific strains such as the poliovirus exist and are critical in preventing severe outcomes such as paralysis [12,16,40,41,42,83,85]. For pediatric patients, the ability to quickly rule out bacterial infections through POC testing also reduces unnecessary antibiotic usage, which is vital for both avoiding antibiotic resistance and ensuring that the patient receives appropriate care [40,41,42,45,82].

### 3.4. Flaviviruses: Dengue, Zika and Yellow Fever Virus

The *Flaviviridae* family contains yellow fever viruses, dengue virus, and Zika viruses, which are leading causes of acute febrile illness worldwide, particularly in tropical and subtropical regions [86,87,88]. Their diagnosis can be challenging due to overlapping symptoms with other viral infections [2,86,89]. New technologies in POC testing methods, such as LFAs and ELISA as discussed earlier, have proven critical in the early detection of dengue, helping to prevent further spread of the virus [86]. POC testing for dengue not only aids in prompt diagnosis but also offers protection by enabling patients to avoid contact with mosquitoes during the viremia phase, thus reducing the risk of viral transmission [90]. In Singapore, the application of POC testing has been particularly effective in reducing unnecessary empirical treatments and hospital admissions [89].

POC testing for dengue virus offers rapid and accessible diagnostics, but it does come with several limitations. One of the key drawbacks is the inability of most POC tests to detect viruses, which is critical for understanding the severity of infection and tracking disease progression [90]. Knowing the VL can help healthcare providers assess how aggressively the virus is replicating and determine the risk of severe complications such as dengue hemorrhagic fever or dengue shock syndrome [86,90]. Additionally, POC tests for dengue often rely on detecting antibodies or antigens, which may not provide an accurate diagnosis during the early stages of infection or in cases where the immune response is delayed [90]. False negatives can occur if the test is performed too early in the infection, before antibodies are detectable, or if a patient has a secondary dengue infection, in which antibody profiles can differ [89]. Moreover, while POC tests are useful for initial diagnosis, they generally lack the capacity for serotyping and cannot differentiate between the four different dengue virus serotypes (DENV-1, DENV-2, DENV-3, and DENV-4) [89]. Individuals previously infected with one dengue serotype face a significantly higher risk of developing severe disease if they become infected with a different serotype [91]. Without the ability to identify the specific serotype, healthcare providers cannot fully assess the risk of severe disease and tailor management strategies accordingly [17,89,90].

Despite limitations, these rapid tests also help identify candidates for Dengvaxia, the first licensed dengue vaccine, which is particularly effective against DENV-3 infections [90,91]. Dengvaxia is recommended for individuals who have previously been infected with dengue, as it offers protection primarily against subsequent infections [91]. It has been approved in several countries, though its use is targeted to those living in high-risk areas due to concerns that it may increase the risk of severe disease in individuals without prior dengue exposure [13,17,89,91]. As the field continues to evolve, improving the precision and scope of POC testing, alongside vaccination efforts, will be critical in controlling dengue outbreaks and minimizing disease impact [91].

## 4. Impact of Viral POC Testing on Surveillance

POC testing has become a critical tool in global health surveillance, especially for managing infectious diseases such as mpox, HBV, respiratory viruses, and dengue. Its ability to provide rapid results supports timely interventions, particularly in resource-limited regions where traditional lab facilities are unavailable. By enabling early detection and real-time monitoring, POC testing enhances outbreak management, helping to curb disease spread and improve public health responses globally [13,14].

### 4.1. Mpox

The recent mpox outbreak has seen significant spread across multiple regions, particularly in parts of Africa, Europe, and North America, with urban centers being hotspots for transmission [49]. Surveillance is crucial to track new cases, monitor the virus’s spread in these affected areas, and prevent further outbreaks, especially in vulnerable communities with limited access to healthcare [49]. According to the WHO’s interim guidance (2022), POC testing plays a vital role in the surveillance and containment of mpox by enabling rapid diagnosis and immediate isolation of infected individuals to prevent further transmission [92]. This is especially critical in regions lacking advanced laboratory facilities, where POC tests offer a simple and accessible method for detecting cases. By facilitating early identification in such settings, POC testing significantly increases the chances of containing outbreaks before they spread widely [51]. Moreover, POC testing enhances real-time surveillance efforts, enabling health authorities to track contacts and monitor the virus’s transmission more effectively [49,51]. This rapid response capacity is essential for public health interventions, helping to curb the spread of mpox and improve overall outbreak management. The availability of affordable, accurate diagnostic tests is crucial not only for preserving global health security but also for controlling emerging or re-emerging infectious diseases.

### 4.2. HBV

Developments in POC testing have greatly improved HBV surveillance efforts, especially in low- and middle-income regions where access to laboratory infrastructure is limited [14]. One of the key advantages of POC testing is its ability to provide same-day results, enabling immediate modifications to antiviral therapy, which can prevent the disease from advancing to more severe stages [12,14]. This rapid response is crucial in improving patient outcomes, especially in preventing complications such as cirrhosis and liver cancer [14]. POC testing also plays a pivotal role in identifying HBV during the window period—a phase early in infection when traditional diagnostic methods may miss the virus due to low levels of detectable surface antigens. By catching cases that would otherwise be missed, POC testing enhances overall surveillance and early intervention [14,58,93].

In addition to improving detection during the window period, POC testing has proven instrumental in identifying HBV “escape variants” [94]. These are mutations in the virus that allow it to evade detection by standard diagnostic methods or even resist antiviral treatments [3,4,5,6,7,8]. Detecting these variants is crucial for effective surveillance, particularly in regions with a high prevalence of HBV, where mutations may spread more rapidly [94]. The ability of POC tests to identify escape variants strengthens public health efforts by ensuring that such cases are not overlooked. Compared with Sanger sequencing, next-generation sequencing (NGS) emerged as a promising tool for identification of mutant viruses [95,96]. NGS turns out to be a useful test that is highly effective in identifying HBV escape variants [97,98]. NGS allows for deep sequencing of the HBV genome, providing comprehensive data on the virus’s genetic makeup. The NGS uses massively parallel sequencing to read the entire HBV genome, enabling the detection of even minor variants within the viral population [97]. By identifying these mutations, NGS can inform clinicians if the patient’s HBV infection is due to a variant that could evade detection by standard diagnostic tests or resist standard antiviral therapies. Moreover, the NGS system is sensitive enough to detect low-frequency variants that may only make up a small proportion of the viral population [97,98]. There are different modalities of NGS chemistries and platform. Among them, Nanopore long-read sequencing only requires small device and has straightforward workflow enabling it to have promise to be developed into portable near-POC rapid sequencing assay for field study, thereby overcoming the limitations of sequencing-based technologies (e.g., requiring expensive instrument and complicated workflows) [99]. For example, Tshiabuila et al. recently developed an Oxford Nanopore technology-based HBV sequencing protocol that is believed to be suitable for genomic surveillance within clinical diagnostic settings in the Western Cape province of South Africa [99].

POC testing for HBsAg has led to increased testing in areas with limited laboratory resources [59]. This expansion in testing coverage is vital for global HBV surveillance, as it ensures that more individuals are identified and treated early, which supports the World Health Organization’s goal of eliminating HBV [62]. By providing real-time data on infection rates and detecting hard-to-find cases, POC testing is a cornerstone in global efforts to monitor, contain, and eventually eliminate HBV [56,58,97,98].

### 4.3. Respiratory Viruses

Influenza POC tests are valuable tools for both diagnosing infections and differentiating between viral subtypes, which is essential for informing vaccine development and deployment. However, these rapid tests come with limitations. During recent influenza outbreaks, some POC tests exhibited moderate inaccuracies, raising concerns about their reliability in certain contexts [100,101]. Such discrepancies can hinder the detection of new strains, such as influenza A (H3N2), where accurate identification is critical for public health responses [101]. Harper et al. emphasized the importance of following test instructions carefully to ensure effective surveillance, especially during seasonal flu epidemics [102]. While POC tests offer convenience and accessibility for surveillance efforts, their results should be supported by additional testing methods—such as viral cultures—to ensure comprehensive monitoring of circulating strains [73]. This is particularly important when differentiating between influenza A and B, as antigen matching is crucial for the formulation of effective vaccines each season [73,101,102].

The COVID-19 pandemic underscored the critical need for scalable and accessible POC testing in global health surveillance [13]. Self-testing tools, such as rapid antigen tests and molecular tests, have played a pivotal role in monitoring the spread of SARS-CoV-2, particularly in areas where gold-standard RT-PCR tests were inaccessible due to cost, logistical challenges, or overburdened healthcare systems [103]. While RT-PCR remains the most accurate method for detecting the virus, as noted by Sakthivel et al., the decentralized and faster deployment of POC tests has been crucial in managing the pandemic on a larger scale. These tests have allowed for widespread surveillance, enabling real-time data collection in community settings, workplaces, and even at-home environments [104].

For ongoing and future surveillance, the adaptability of POC tests is vital, especially as the virus continues to evolve [12,104]. The emergence of new variants, driven by mutations in the viral genome, necessitates constant updates to NAATs and antigen-based tests to maintain their accuracy and effectiveness [103]. Quality control in the development and deployment of these tests will be essential to ensure reliable detection and accurate tracking of viral variants [103,104]. The ability of POC tests to quickly evolve in response to these genetic changes will be critical not only for tracking subsequent waves of COVID-19 but also for monitoring other respiratory pathogens that may arise in the future. This adaptability will ensure that POC testing remains a cornerstone of global health surveillance and pandemic preparedness [13,104]. Furthermore, keeping updating primers and probes with the pace with viral evolution is important. For example, pan-sarbecoviruses-conserved regions in the Envelope gene of SARS-CoV and SARS-CoV-2 have been used as targets for multiple RT-PCR assays to detect SARS-CoV-2 since the onset of COVID-19 pandemic [105]. Liu et al. [105] demonstrated that SARS-CoV-2 variants of concern (VOCs) have accumulated significant mutations in Envelope gene that may affect Envelope protein stability and diagnostic RT-PCR assays. Liu et al. performed a comprehensive genetic analysis on the conservation and diversity of the Envelope gene of SARS-CoV-2 and its VOCs in comparison with other human coronaviruses [105]. They identify conserved central region in the Envelope gene that may be suitable for primers/probes design for SARS-CoV-2 or pan-HCoVs screening and diagnostic assays [105].

Finally, many enteroviruses, particularly Enterovirus D68, pose a significant public health threat, especially during epidemic seasons, where they primarily affect children and cause severe respiratory illnesses [82]. Antigen testing and virus assessments are crucial tools for monitoring these infections. Real-time PCR has been identified as particularly effective in analyzing outbreaks of Enterovirus D68, enabling swift public health interventions, preventing viral transmission, and mitigating severe outcomes [85,106]. This rapid response capability is essential for containing outbreaks before they escalate, allowing health authorities to organize preventive measures more efficiently [75,106].

POC testing plays a key role in the broader surveillance of enteroviruses, especially within national systems such as the National Respiratory and Enteric Virus Surveillance System (NREVSS), which monitors these viruses on a large scale [82,107]. However, while POC testing enhances surveillance efforts, it can contribute to under-reporting during non-epidemic seasons, highlighting the need for continuous and effective community-level monitoring [107]. Sustainable surveillance should be linked to real-time diagnostics, clinical case descriptions, and population-based epidemiology to ensure that enteroviruses are tracked comprehensively throughout the year [107]. This comprehensive approach is crucial for maintaining preparedness, identifying potential outbreaks early, and protecting public health from the evolving threat of enteroviruses [76,106,107].

### 4.4. Flaviviruses: Dengue, Zika and Yellow Fever Virus

Given the significance of *Flaviviridae* members in public health, surveillance and virology, POC testing has been pivotal in flaviviral surveillance, particularly in areas heavily affected by the virus [86,108,109]. Raafat et al. highlighted the effectiveness of POC PCR tests in providing timely results in low-income regions, where traditional laboratory infrastructure is often lacking [89,109]. These tests are critical for field implementation, allowing for real-time diagnostics and immediate public health responses. However, one limitation of POC testing for dengue is its inability to differentiate between the various serotypes of the virus, which is crucial for vaccine selection and disease management strategies [94,109].

In addition to basic diagnostics, POC testing offers a more robust tool for tracking disease progression and immune response outcomes [90]. Such integration helps calculate both initial and subsequent immune responses in the population, which is essential for informing public health measures and interventions in dengue-endemic regions [90,109]. Although there are challenges related to the sensitivity and specificity of some POC tests, particularly in detecting all dengue serotypes, they remain indispensable in real-time surveillance and the control of dengue outbreaks [86]. By enabling rapid identification and response, POC testing continues to be a key tool in mitigating the spread of the virus and managing public health efforts in affected areas [86,89,90,109].

## 5. Conclusions

In conclusion, this review highlights the critical role that POC diagnostics and VL testing play in the management and surveillance of VPVIs such as mpox, hepatitis B, influenza, dengue, and COVID-19. The advances in POC technologies, including molecular assays, biosensors, and CRISPR-based systems, have improved the speed, accuracy, and accessibility of viral detection, enabling healthcare providers to make timely and informed treatment decisions. Furthermore, the integration of smartphone-based diagnostics has expanded the reach of these technologies to resource-limited settings, offering real-time diagnostic capabilities that support global health initiatives. As VPVIs continue to pose public health challenges due to breakthrough infections and emerging strains, the relevance of these POC tools in ensuring effective patient care and outbreak control is undeniable.

The broader implications of these advancements extend beyond individual patient outcomes, as they are essential for enhancing global health surveillance and response efforts. By providing immediate and accurate data on viral infections, POC diagnostics contribute to more effective public health interventions, especially in areas with limited healthcare infrastructure. The rapid detection and monitoring capabilities offered by these technologies are particularly valuable in preventing widespread transmission, guiding vaccination strategies, and ensuring the timely allocation of resources during outbreaks. As POC technologies continue to evolve, further research and optimization will be key to maximizing their potential in improving healthcare delivery and mitigating the impact of viral infections on a global scale.

## 6. Perspectives

The future of POC diagnostics for VPVIs is promising, with advancements in technologies like CRISPR-based assays, biosensors, and smartphone-integrated devices are expected to transform public health and patient care. These innovations offer rapid, sensitive, and specific on-site testing, which is crucial for timely outbreak response, personalized treatment decisions, and health monitoring in resource-limited settings. The integration of POC diagnostics with digital health platforms will further amplify their impact by enabling real-time data sharing and surveillance, essential for tracking disease spread and guiding public health interventions. Smartphone-based devices, in particular, offer unprecedented scalability and accessibility, making POC testing a key tool for both individual care and global health surveillance.

Looking ahead, the optimization of POC testing will focus on increasing affordability, ease of use, and regulatory compliance to enable widespread, equitable deployment. Pandemics and epidemics continue to be a significant issue in many areas with poor access to healthcare. It is vital that resources are spread to these areas. As these technologies evolve, they promise to fortify healthcare systems worldwide by supporting rapid diagnostics, enhancing global surveillance, and improving the overall management of viral infections at both individual and community levels.

## Data Availability

Not applicable.
